# Specific Visualization of Nitric Oxide in the Vasculature with Two-Photon Microscopy Using a Copper Based Fluorescent Probe

**DOI:** 10.1371/journal.pone.0075331

**Published:** 2013-09-23

**Authors:** Mitrajit Ghosh, Nynke M. S. van den Akker, Karolina A. P. Wijnands, Martijn Poeze, Christian Weber, Lindsey E. McQuade, Michael D. Pluth, Stephen J. Lippard, Mark J. Post, Daniel G. M. Molin, Marc A. M. J. van Zandvoort

**Affiliations:** 1 Department of Genetics & Cell Biology-Molecular Biology, Cardiovascular Research Institute Maastricht (CARIM), Maastricht University, Maastricht, The Netherlands; 2 Department of Physiology, Cardiovascular Research Institute Maastricht (CARIM), Maastricht University, Maastricht, The Netherlands; 3 Department of Pathology, Cardiovascular Research Institute Maastricht (CARIM), Maastricht University, Maastricht, The Netherlands; 4 Department of Surgery, Nutrition and Toxicology Research Institute Maastricht (NUTRIM), Maastricht University, Maastricht, The Netherlands; 5 Institute for Molecular Cardiovascular Research (IMCAR), RWTH University Aachen, Aachen, Germany; 6 Department of Cardiology and Angiology, Medizinischen Fakultät der Westfälischen Wilhelms-Universität, Münster, Germany; 7 Institute for Cardiovascular Prevention, Ludwig-Maximilians-University (LMU), Munich, Germany; 8 Department of Chemistry, Massachusetts Institute of Technology (MIT), Cambridge, Massachusetts, United States of America; Center for Cancer Research, National Cancer Institute, United States of America

## Abstract

To study the role and (sub) cellular nitric oxide (NO) constitution in various disease processes, its direct and specific detection in living cells and tissues is a major requirement. Several methods are available to measure the oxidation products of NO, but the detection of NO itself has proved challenging. We visualized NO production using a NO-sensitive copper-based fluorescent probe (Cu _2_FL2E) and two-photon laser scanning microscopy (TPLSM). Cu _2_FL2E demonstrated high sensitivity and specificity for NO synthesis, combined with low cytotoxicity. Furthermore, Cu _2_FL2E showed superior sensitivity over the conventionally used Griess assay. NO specificity of Cu _2_FL2E was confirmed *in vitro* in human coronary arterial endothelial cells and porcine aortic endothelial cells using various triggers for NO production. Using TPLSM on *ex vivo* mounted murine carotid artery and aorta, the applicability of the probe to image NO production in both endothelial cells and smooth muscle cells was shown. NO-production and time course was detected for multiple stimuli such as flow, acetylcholine and hydrogen peroxide and its correlation with vasodilation was demonstrated. NO-specific fluorescence and vasodilation was abrogated in the presence of NO-synthesis blocker L-NAME. Finally, the influence of carotid precontraction and vasorelaxation validated the functional properties of vessels. Specific visualization of NO production in vessels with Cu _2_FL2E-TPLSM provides a valid method for studying spatial-temporal synthesis of NO in vascular biology at an unprecedented level. This approach enables investigation of the pathways involved in the complex interplay between NO and vascular (dys) function.

## Introduction

Endogenously produced vascular nitric oxide (NO) affects important biological processes such as platelet and leukocyte adhesion, smooth muscle cell (SMC) migration, and endothelial regeneration in blood vessels [1,2,3,4]. Moreover, the regulation of blood flow through induction of vasodilation is a major function of endothelial-derived NO. Cellular NO is produced by three different enzymes (i.e. iNOS, eNOS, nNOS) [3], of which endothelial nitric oxide synthase (eNOS), specifically expressed in endothelial cells (ECs), is essential for physiological NO (order of nanomolar range) [5,6] production in healthy blood vessels. In response to increased shear stress, eNOS is activated in the endothelium [2,3], with subsequent production of NO. NO then diffuses to the neighboring SMCs, where it induces vasodilation through SMC relaxation and subsequently increases vessel lumen diameter [4,5] and blood flow. Abrogation of NO production in dysfunctional endothelium is involved in numerous acute and chronic cardiovascular diseases such as hypertension and atherosclerosis [3,6]. The direct and specific detection of NO in living cells and tissues is a major, hitherto unmet, requirement for investigating the role and (sub) cellular NO constitution in various disease processes.

Ongoing research has been aimed at detecting and quantifying physiological NO levels [2], but the high diffusibility and short half-life (3-16 sec.) of NO complicate real time detection [7,8,9]. Hence, little is known about the time course and diffusion profile of endogenously produced NO. Several chemical methods are available to measure the oxidation products of NO, such as nitrite or nitrate, but the detection of NO itself has proved challenging. We used fluorescent probe-based imaging methods to study NO dynamics. The high sensitivity, spatial resolution, and experimental feasibility make fluorescent-based methods the preferred imaging modality [6,7,8]. An added advantage of this strategy is that structural and functional imaging can be executed simultaneously [5,10].

**Figure 1:**
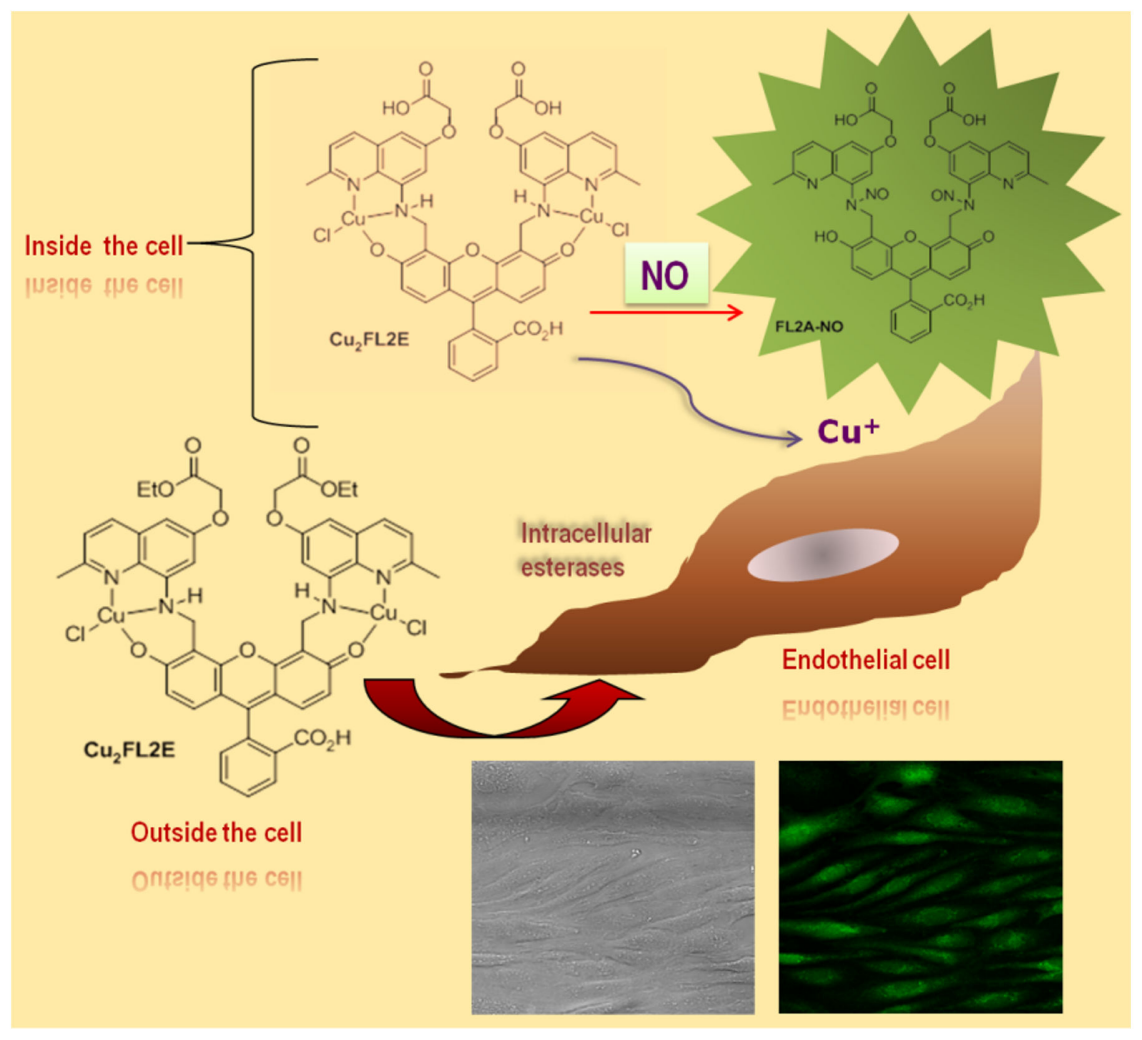
Scheme of NO detection by Cu _2_FL2E in endothelial cells, showing the probe is trapped in the cell via hydrolysis of the pendant ester groups by intracellular esterases to give Cu _2_FL2A since the ester (E) is hydrolyzed to the acid (A).

In the present study, we evaluated the feasibility and characteristics of a previously defined specific, cell-trappable, copper-based fluorescent NO probe (Cu _2_FL2E) for vascular NO analysis both *in vitro* and *ex vivo*. Cu _2_FL2E, developed by McQuade L.E. et al. [11], has highly desirable properties. It is non-toxic and readily internalized by cells *in vitro*. Moreover, it reacts with NO directly and specifically rather than with its oxidation products. Upon reaction of Cu _2_FL2E with NO, Cu(II) is reduced to Cu(I) with concomitant formation of highly fluorescent, *N*-nitrosated FL2E-NO [11,12,13]. These characteristics of Cu _2_FL2E make it a useful intracellular sensor for NO. Furthermore, cell trappability of the probe is imparted when the pendant ester groups are hydrolyzed by intracellular esterases, yielding Cu _2_FL2A, the negatively charged acid (Figure **1**).

In this paper we establish Cu _2_FL2E as a valuable tool for direct and specific imaging and visualization of NO in ECs *in vitro* and, in conjunction with TPLSM, *ex vivo* in intact vessels with high spatio-temporal accuracy and large penetration depth [5,10]. We show that this methodology allows for relative quantification of NO and exploration of NO-mediated vasomotor response *ex vivo*.

## Materials and Methods

### 1: Ethical statement

The local ethics committee (FHLM, Maastricht University) on use of laboratory animals approved all experiments. Procedures were in accordance with institutional guidelines. For *ex vivo* experiments euthanasia was performed by applying a mixture of CO_2_ and O_2_, after which arteries were isolated. Carotid artery segments (common part) and aorta segments were excised from 20-22 weeks old C57BL6/J (n=6) mice (Charles River, Maastricht, the Netherlands). For isolation of PAECs, Dutch Landrace pigs of 40 to 50 kg were euthanized using pentobarbital. Other cells were commercially obtained [Lonza].

### 2: Chemical Reagents

*S*-nitroso-N-acetyl-D,L-penicillamine (SNAP) [Sigma Aldrich], acetylcholine (ACh) [Sigma Aldrich], L-N^G^-nitroarginine methyl ester (L-NAME) [Sigma Aldrich], phorbol 12,13 dibutyrate ester (PE) [Sigma], endothelial cell medium (ECM) [Lonza], Hanks balanced salt solution (HBSS) [Lonza], Phosphate buffer solution (PBS) [Lonza], propidium iodide (PI) [Invitrogen], 4',6-diamidino-2-phenylindole (DAPI) [Invitrogen], hydrogen peroxide [Merck], noradrenaline (NA) [Sigma].

### 3: Spectroscopic Materials and Methods

Spectroscopic measurements were made on a Nanodrop (ND3300, Thermo Scientific) fluorescence spectrometer. Cu _2_FL2E was used for determining spectral properties, SNAP was used to release NO in the solution, PBS was used for dissolving Cu _2_FL2E to make solutions of suitable concentration, and hydrogen peroxide was used to determine change in spectral characteristics of Cu _2_FL2E on reacting with this reactive oxygen species. Copper (II)-chloride dihydrate (99%, Sigma Aldrich) stock solutions of 1 mM were prepared in Millipore water. Stock solutions of 1 mM ligands (FL2E) were prepared in DMSO. Probe (Cu _2_FL2E) concentrations were generated by combining stock solutions of CuCl_2_ and FL2E in a 2:1 ratio. Cu _2_FL2E was dissolved in PBS to get the desired concentration. Cu _2_FL2E (2 µM) was allowed to react with the NO-releasing chemical agent SNAP at pH 7. Replicate fluorescence measurements were taken at 1 min for 2 µL of solution of Cu _2_FL2E probe with or without SNAP. For the spectral measurements, a white LED (460-650 nm) was used for Cu _2_FL2E based on the detected maximum absorption.

### 4: Cytotoxicity assay

HCAECs were seeded into 24-well plates (500 µL total volume/well, 5000 cells/cm^2^) in complete ECM and incubated at 37°C with 5% CO_2_ for 72 h until confluent. The medium was replaced and cells were incubated with or without Cu _2_FL2E (2 µM-200 µM) in OptiMEM media for 1 h in triplicate. The medium was removed and replaced with HBSS and incubated with double stains; propidium iodide (1.5 µM) for dead/dying cells and DAPI (0.1 µM) for nuclear staining. The cells were then incubated for 30 min in the dark. Washing and imaging was done in HBSS. The percentages of cell survival values were calculated from 5 different images as ratio of PI/DAPI of nuclear staining. 5 images per condition were made with a fluorescence microscope. Total amount of cells (DAPI-positive) and PI-positive (leaky, thus dead or damaged) cells were counted. Ratios were averaged.

### 5: Griess assay

Cells were seeded in a 6-wells plate and grown until confluent. Medium was removed and 1.5 mL of fresh culture medium with or without 150 mM H_2_O_2_ was added and 200 µL was sampled at t = 0, 1, 6, and 24 h. Medium was centrifuged using 10,000 Da MWCO polysulfone filters (Sartorius) and supernatant was analyzed using the Total Nitric Oxide Assay Kit (Assay Designs) [14].

### 6: Cell cultures and imaging materials and methods

Porcine aortic endothelial cells (PAECs) were isolated from thoracic aorta [15]. PAECs and human coronary artery endothelial cells (HCAECs; Lonza) were cultured in EGM-2MV medium (ECM; Lonza). For imaging studies, cells were plated onto poly-d-lysine coated 6 wells plates, and cultured until confluency was reached at 37°C with 5% CO_2_. To study NO production, the Cu _2_FL2E probe was added in a concentration of 20 µM (40 µM CuCl_2_ + 20 µM FL2E) in the case of PAECs and HMVECs, and 2µM (4µM CuCl_2_ + 2µM FL2E) in the case of HCAECs, diluted in OptiMEM (Invitrogen). Cells were co-incubated with Cu _2_FL2E and stimulus (10 µM ACh [Sigma] [16] or 150 µM H_2_O_2_ [Merck]) for 45 min and washed three times with HBSS prior to imaging. For inhibitor studies, HCAECs were preincubated with 100 µM L-NAME for 1 h prior to addition of Cu _2_FL2E and stimulus. Images were acquired on an inverted fluorescent microscope (Leica DMI3000B) equipped with FITC filter and a DFC350 FX camera (Leica). Images were taken after removing the DMEM media and washing the cells with Hanks buffered salt solution (HBSS). Additionally, two-photon microscopy was performed (see below) in some cases. All fluorescent images were corrected for background.

### 7: Tissue preparation

Segments of murine common carotid arteries (length ~ 6-8 mm) were explanted and freed of adipose and connective tissue and carefully handled, only at their outer ends without stretching them, to keep them viable. To avoid contact with air, they were kept moist during the whole preparation procedure. Until further processing, arteries were stored (maximum 30 min) at 4°C in HBSS, pH 7.4, containing: NaCl 144mM, HEPES 14.9mM, glucose 5.5mM, KCl 4.7mM, CaCl_2_ 2.5mM, KH_2_PO_4_ 1.2mM, and MgSO_4_ 1.2mM.

### 8: Mounting procedure

The murine common carotid arteries were explanted and mounted on a perfusion chamber [5] filled with 10 ml ECM (37°C). The artery was mounted on two glass micropipettes (tip diameters 120-150 µm for carotid arteries) and residual luminal blood was carefully removed by gently flushing with HBSS. To correct for the shortening of the artery during isolation, a transmural pressure of 100 mmHg was applied (using a modified Big Ben sphygmomanometer, Riester, Germany) and the distance between the two pipettes was adjusted until the mounted artery was straight. After this length adjustment, transmural pressure was set at 80 mmHg to mimic physiological conditions. All experiments were performed at 37°C (Linkam scientific instruments MC60 heating stage, UK) in the absence of luminal flow. Imaging was performed in vessels at a transmural pressure of 80 mmHg and was restricted to the central portion of the vessel segment. Only weak auto fluorescence could be observed with TPLSM. After that the vessel was incubated with Cu _2_FL2E probe (20 µM) for 5 min with luminal flushing of the probe. After washing excess probe from the solution and luminal washing, it was again imaged with TPLSM. The measurements on vessels without pre-contraction the vessel was then incubated with ACh (10 µM) or H_2_O_2_ (150 µM) for 45 min and then washed and imaged. In case of measurements with L-NAME (100 µM) it was pre-incubated for 1 h. For experiments with pre-contraction and relaxation of vessels, NA (10 µM) was added, followed by immediate addition of ACh, inducing signal within 2 minutes. For flow experiments, laminar flow (2.1Pa) shear stress, mimicking the blood flow was applied as stimulus for NO production for 45min. After that the vessel was incubated with Cu _2_FL2E probe (20 µM) for 5 min with luminal flushing of the probe. After washing excess probe from the solution and luminal washing, it was again imaged with TPLSM. Compared to carotid arteries, aorta was more difficult to mount and pressurize for our experiments because of its numerous side branches; nonetheless it was mastered successfully by ablation (burning) of side branches and NO signal was sub-cellularly studied along with vasomotor response. In case of denudation of endothelium from carotid artery, a hair was used to insert at the luminal side in the excised carotid artery and disrupt the endothelium. NO signal was sub-cellularly studied along with vasomotor response.

### 9: Wire myography

2mm segments of mouse carotid arteries were mounted between two stainless steel wires (40 µm in thickness) connected to a displacement device and an isometric force transducer (DSC6; Kistler Morse, Seattle, WA), respectively, in organ chambers (DMT, Aarhus, Denmark) filled with KRB solution at 37°C, and they were aerated with 95% O_2_, 5% CO_2_. The segments were progressively stretched to the diameter at which their contractile response to 10 µM noradrenaline was maximal. They were relaxed by administration of 10 µM ACh. Experiments were repeated after addition of Cu _2_FL2E probe (20 µM) for 5 min in the chamber and washing.

### 10: Two-photon imaging

The perfusion chamber with artery or a 6-well plate with cultured endothelial cells was positioned on a Leica ultrafast TCS SP5 multiphoton microscope. The microscope integrated with DM6000 CFS (Confocal fixed stage) system, DFC360FX camera system and AOBS (Acousto optical beam splitter) [Leica, Manheim, Germany] was used. The excitation source was a Chameleon Ultra Ti: Sapphire laser (690-1040 nm) (Coherent Inc. Santa Clara, CA, USA), tuned and mode-locked at 800 nm and producing light pulses of repetition rate 80 MHz with duration of about < 200 femtosecond. The pulses reached the sample through the Leica HCX APO L 20x/1.0W microscope objective (20 X, water dipping, numerical aperture 1.0, working distance 2 mm, access angle 39 degrees), connected to an upright Leica DM6000 CFS microscope. An optical zoom in the scan head achieved further magnification. Super Z-galvo was used for fast and accurate XYZ and XZY scan mode. External detectors were used for collecting emitted lights from the sample. When desirable the fluorescence was detected by one independent photomultiplier tube (PMT) for the green wavelength region (508-523 nm). Images of 512 × 512 pixels were obtained. For quantification purposes, the intensities in the green channel were used. The excitation wavelength of 800 nm was chosen since we found that at this wavelength both traps and adducts were effectively excited. No additional image processing was performed. For imaging of the endothelial cells and carotid arteries, an imaging speed of 50 Hz was used to improve signal-to-noise ratio. To prevent photochemical and thermal damage to the arteries, laser power was kept as low as possible [5]. Images were recorded in the XY-plane. Fluorescence images were taken after removing the EGV-2M media and by washing the cells or flushing the artery with Hanks buffered salt solution (HBSS). Inaccurate alignment of the pipettes in the perfusion chamber usually caused imaging of the artery in a slightly oblique plane. Series of XY-images at successive depths (Z-stack) were collected for reconstruction of 3D images. Luminal diameters were obtained from XZ images. To obtain XZ images with square pixels, z-step distance was equal to the pixel dimensions in XY-direction. In case of vessels, the scanning takes place in the direction from adventitial layer to the intimal layer, making optical sections and compiling in Z-stacks.

### 11: Image analysis

Images were analyzed using LASAF acquisition software (Leica, Manheim, Germany). 3D reconstructions of images were made using Image-Pro Plus 6.3 software (Media Cybernetics Inc., USA). NO mediated vasomotor responses, i.e. functionality of arteries, were determined in three carotid arteries and measured as changes in luminal diameter in XZ scans of the vessel. Signal quantification was done by using LASAF software to check florescence intensity (au) in different regions of interest.

### 12: Statistics

Results were presented as mean ± standard deviation and were tested for significance using the t-test (non-parametric test for two independent groups). A value of P < 0.05 was considered to be statistically significant. Linear regression analysis was performed to assess best-fit relation, slope and correlation coefficient. R square value near to 1.0 shows best-fit predictability. All statistical analyses were performed using GraphPad Prism software (GraphPad Software Inc., USA)

## Results

### Cu _2_FL2E is highly sensitive and specific for NO detection

**Figure 2:**
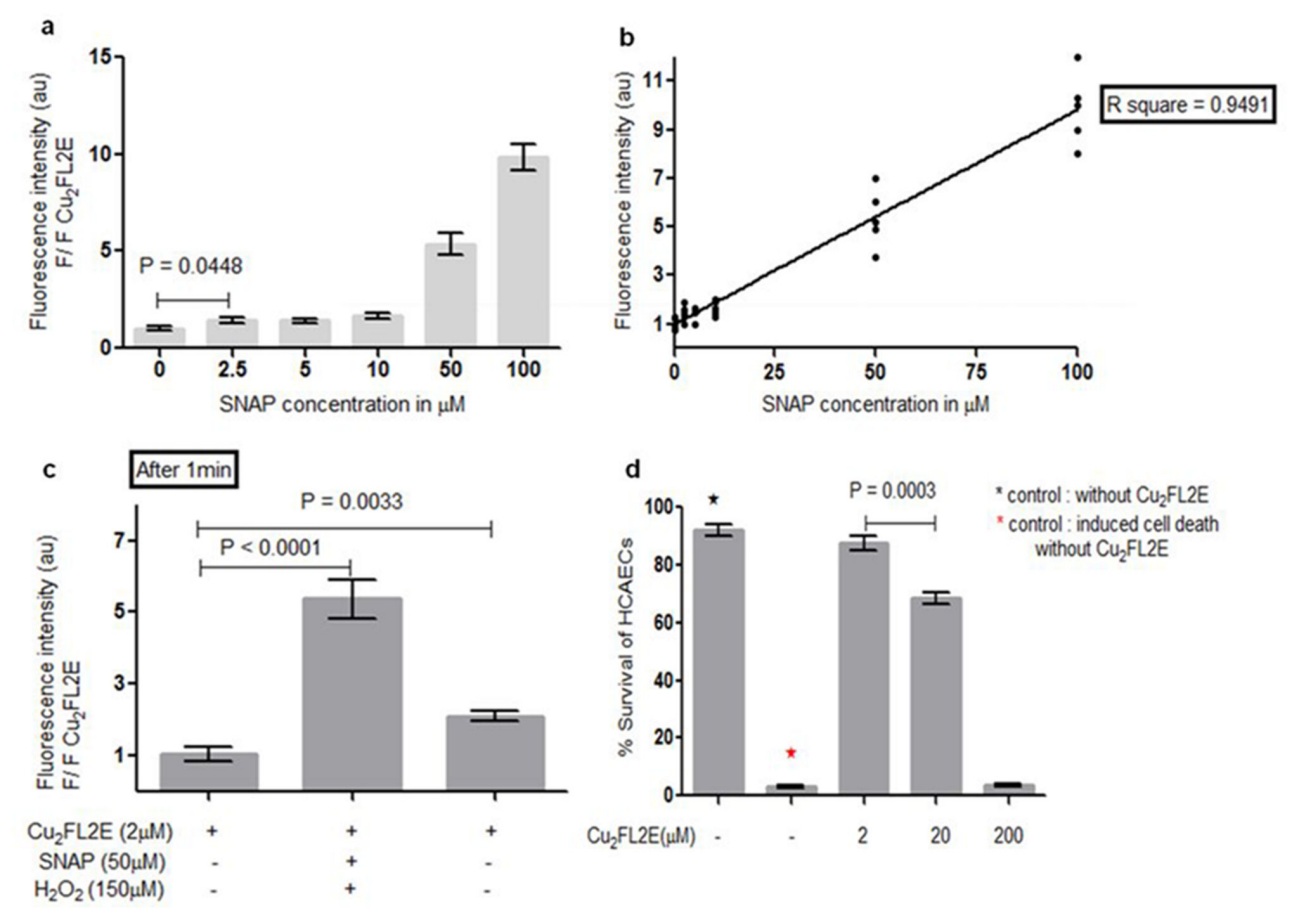
Sensitivity and specificity of Cu _2_FL2E. (**a**) Fluorescence response of Cu _2_FL2E (2 µM) to various concentrations of NO after 1 min of SNAP administration. n = 5 for each concentration, (**b**) Linear regression curve plotted from (a), (**c**) Fluorescence response of Cu _2_FL2E to NO (50 µM SNAP in PBS at 37°C, pH 7.4) and H_2_O_2_ (150 µM). The spectra were obtained 1 min after SNAP addition n = 5. Error bars indicate s.d., (**d**) Cytotoxicity assay with different concentrations Cu _2_FL2E.

To underscore the sensitivity of Cu _2_FL2E to NO, we started with its spectroscopic behaviour in response to various concentrations of SNAP as NO donor [17] in phosphate buffer solution (PBS). We refer to the discussion for a further motivation and justification of this choice. At a concentration of 100 µM in PBS at pH=7.4 and 37°C, SNAP produces 1.4 µM NO per min. [17,18]. The same conditions were used in our study. For Cu _2_FL2E (2 µM) the lowest detectable concentration of SNAP after 1 min was found to be 2.5 µM, corresponding to around 35 nM NO, (Figure **2a**). The concentration dependence of Cu _2_FL2E to SNAP was fit linearly, and the concentration dependence (Figure **2b**) of Cu _2_FL2E exhibited a high correlation coefficient (R^2^ = 0.95), and high steepness (Slope = 0.088 ± 0.004x), indicating good fit and high sensitivity response, respectively.

Additionally, although the specificity of Cu _2_FL2E for NO over H_2_O_2_, HNO, NO_2_^-^, NO_3_^-^ and ONOO^-^ has been demonstrated previously [11,13], we also compared the reaction of Cu _2_FL2E to SNAP-induced NO production (50 µM) with that to H_2_O_2,_ (150 µM). This is especially relevant because the latter is used in our study as one of the stimuli for NO production in cells and vessels [19,20,21]. Figure **2c** shows that, 1 min after SNAP addition, Cu _2_FL2E (2 µM) strongly reacts with NO to produce a 5-fold increase in fluorescence intensity compared to background (p-value < 0.0001). In contrast, the addition of H_2_O_2_ generated a much smaller (2-fold) increase in fluorescence intensity (p-value = 0.003). These data compare well with data of McQuade L.E. et al. (supplementary information) [11]. We consider that H_2_O_2_ can indeed slightly activate the probe but the specificity for NO is evident, as the NO-effect is 3-fold higher than the response to H_2_O_2_.

Finally, to study the cytotoxicity of Cu _2_FL2E, we loaded human coronary endothelial cells (HCAECs) with various concentrations of Cu _2_FL2E. There was undetectable cytotoxicity at 2-20 µM Cu _2_FL2E, the regular concentration for cellular imaging of NO (Figure **2d**). Cytotoxicity arose only at concentrations above 20 µM.

### NO production can be imaged specifically *in vitro* in endothelial cells with Cu _2_FL2E

**Figure 3:**
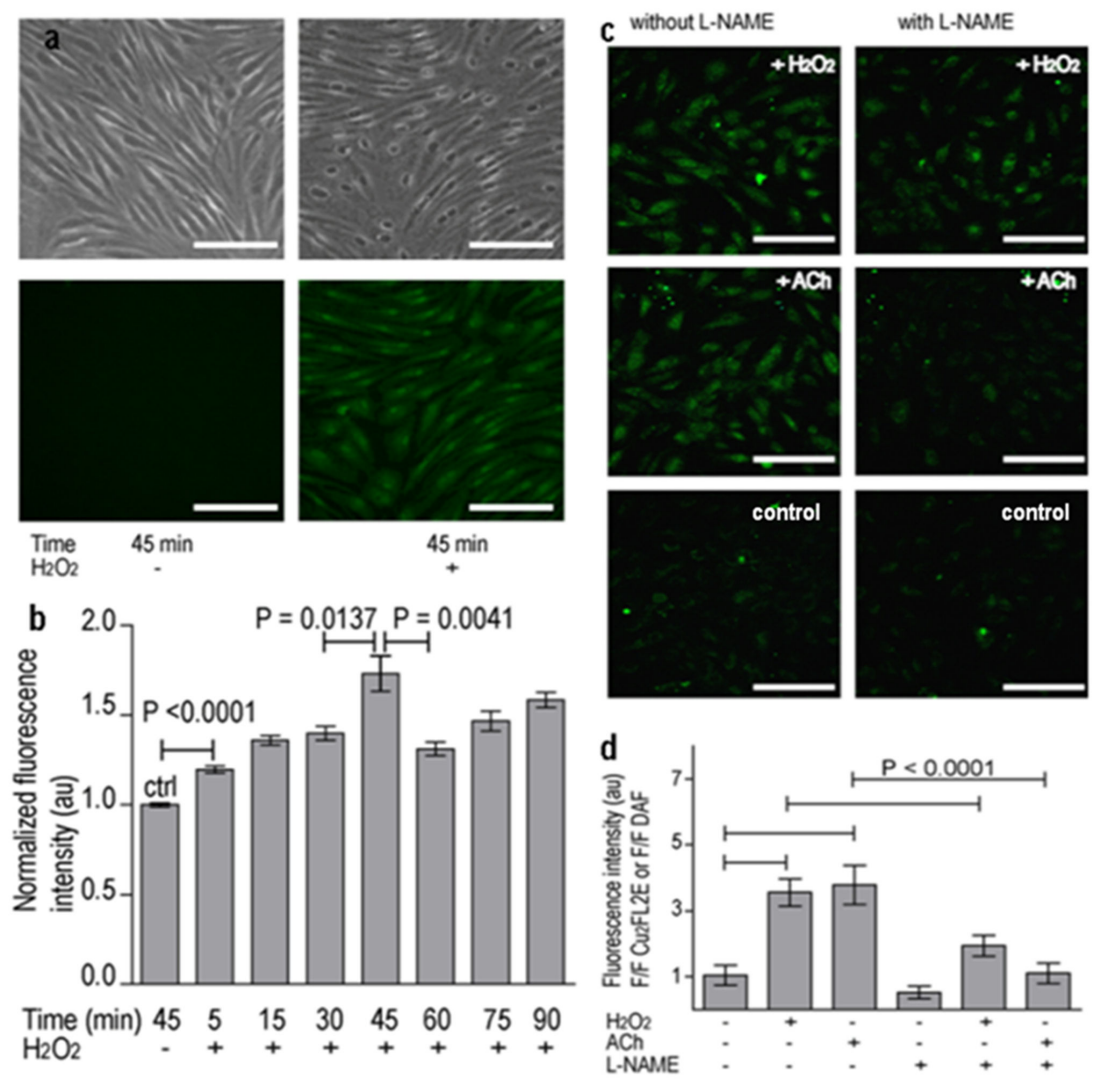
Detection of NO with Cu _2_FL2E produced by endothelial cells *in* *vitro*. (**a**) NO detection in porcine aortic endothelial cells (PAECs); Left: 45 min incubation of Cu _2_FL2E (20 µM). Right: 45 min incubation of Cu _2_FL2E (20 µM) and H_2_O_2_ (150 µM). Top: bright-field images of cells. Bottom: fluorescence images of cells. Scale bar is50 µm. (**b**) Quantification of fluorescence intensity plotted against incubation time. (**c**) Detection of NO with Cu _2_FL2E in HCAECs cells, with or without NO-inhibitor (L-NAME). Shown are the fluorescence images after 45min co-incubation of the probe (Cu _2_FL2E =2 µM) with H_2_O_2_ (150 µM), L-NAME (100 µM), and/or ACh (10 µM) according to scheme. Scale bar is 75 µm. (**d**) Quantification of fluorescence intensity from (c) plotted against each condition mentioned in (c) (n = 5). Error bars indicate s.d.

The ability of Cu _2_FL2E to detect NO produced in different EC types under the influence of various stimuli was investigated. Firstly, Cu _2_FL2E-loaded (20 µM) porcine aortic endothelial cells (PAECs) were stimulated with H_2_O_2_ (150 µM) and the time-dependent fluorescence enhancement was monitored. It is known that H_2_O_2_-induced NO synthesis under these conditions in ECs proceeds via activation of eNOS through coordinated phosphorylation and dephosphorylation of eNOS amino acid residues between 5 to 45 min [22]. We followed NO production over 90 minutes following H_2_O_2_ supplementation. In agreement with the NO-genesis profile, we detected NO production by a rise in fluorescence intensity above background in ECs, starting already 5 min after H_2_O_2_ exposure. After 45 min, the fluorescence intensity reaches a plateau (Figure **3a& b**). Indeed, because Cu _2_FL2E reacts irreversibly with NO, the intensity of the fluorescence signal initially increases as more NO is produced [11,13]. Eventually, however, a signal plateau is reached when NO synthesis is reduced over time or when the entire probe has reacted with NO. However, the minor drop in fluorescence seen at 60 min when compared with 45 min in Figure 3b might be due to photobleaching or chemical quenching. NO detection with Cu _2_FL2E was considerably faster (starting after 5 min) than the traditional Griess assay [14], which required hours of stimulation for significant detection (data not shown).

Next, the reaction of HCAECs to the addition of either H_2_O_2_ (150 µM) or Ach (10 µM) as NO generator was tested at 45min after stimulation, both without and in the presence of L-*N* ^G^-nitroarginine methyl ester (L-NAME), a widely used nitric oxide synthesis inhibitor [7,17,19,20,23]. Based on past experience, we used low-mid passage HCAECs (passage <6) for cellular studies because cells cultured at high passage number (above passage 9) lose their ability to respond to ACh-induced NO synthesis. As expected, the fluorescence signal in the presence of stimulus and inhibitor was significantly weaker (2 to 3-fold, p-value < 0.0001) than in the presence of stimulus alone for both stimuli (Figure **3c& d**). These data again demonstrate the NO-specific response of Cu _2_FL2E and its validity for studying cellular NO synthesis.

### NO production is functionally tested and can be visualised *ex vivo* in precontracted murine carotid arteries with Cu_2_FL2E

In most physiological experiments, ACh is added after pre-contraction of the vessel. To demonstrate that Cu _2_FL2E and its NO scavenging capacity did not affect the vasomotor function of the carotid arteries, the influence of Cu _2_FL2E on vascular contractility was analyzed in the myograph. In this experimental setup, the excised and unlabeled murine carotid artery was precontracted with NA (10 µM) and, thereafter, stimulated with ACh (10 µM) to induce the vasomotor response in the presence or absence of Cu _2_FL2E (20 µM). Note that for the relaxation of carotids a rather high concentration of ACh [16] is needed and the changes in diameter are expectedly small. The results (not shown) revealed no differences in percentage of diameter change and time-scale of relaxation in the presence of Cu _2_FL2E.

**Figure 4:**
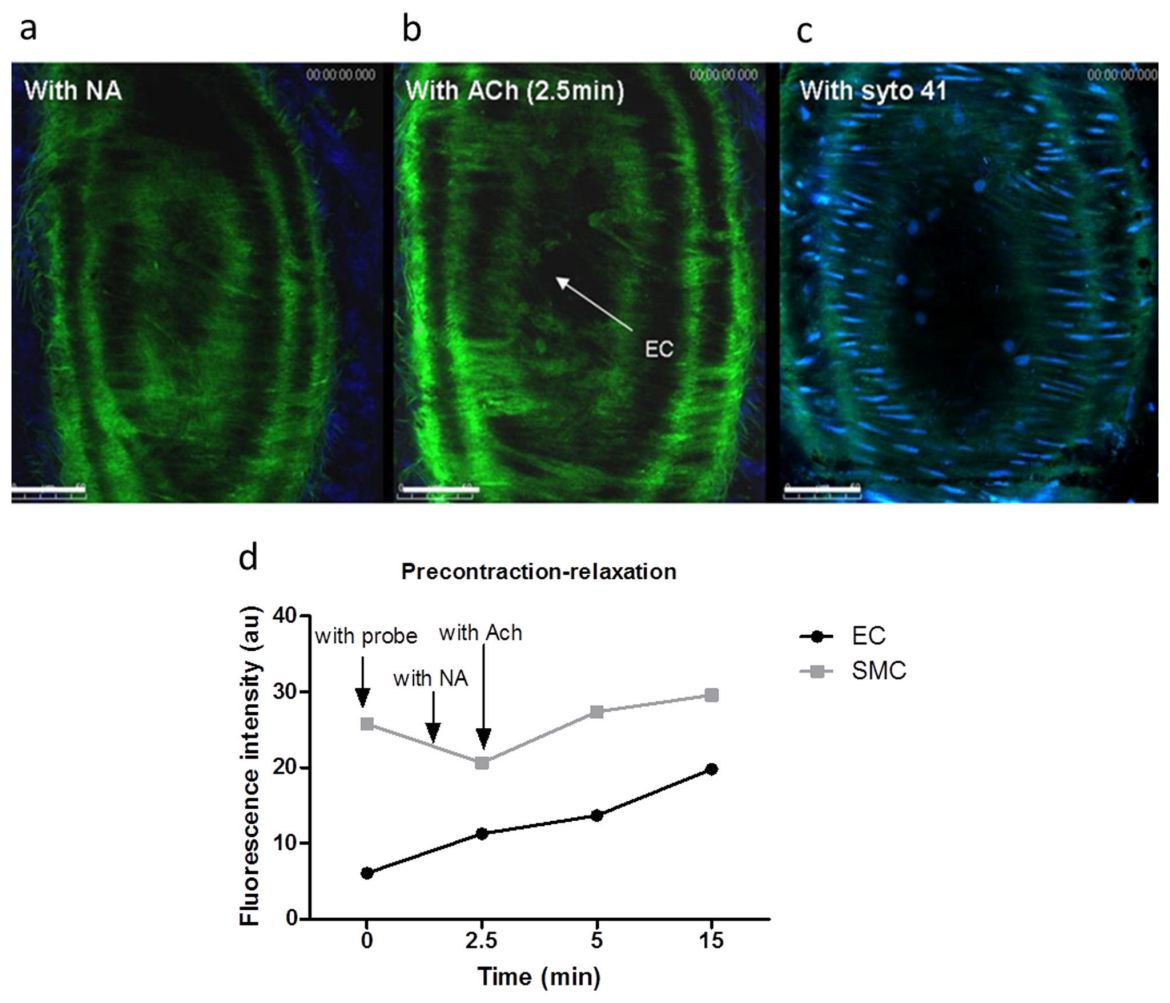
Detection of NO produced in explanted murine carotid arteries *ex* *vivo* using Cu _2_FL2E (20 µM) after precontraction. (**a**) Detection of NO in response to NA (ECs and SMCs are not apparent), (**b**) Detection of NO in post NA and ACh stimulation (2.5min) (ECs and SMCs are apparent), (**c**) Syto 41 staining of nucleus of ECs and SMCs, (**d**) plot of fluorescence intensities of the ECs and SMCs (from carotid artery) measured with NA and ACh stimulation for 15min.

Next, to determine the time curve for ACh-induced NO fluorescence signal, murine carotid arteries were explanted and mounted in a custom-made perfusion chamber [5,10]. Weak, but clearly distinguishable autofluorescence was detected from the elastin fibres of the vessel and was used to locate relevant vascular layers. To reach pre-contraction, NA was applied as vasoconstrictor after Cu _2_FL2E pre-incubation. The autofluorescence was independent of the presence of NA and Cu _2_FL2E. Individual SMCs and ECs could not be identified at the starting point of analysis (i.e. in absence of the probe, in the presence of the probe but without external stimulus, or in the presence of NA) due to lack of sufficient cellular autofluorescence or basal NO signal above threshold of detection, respectively. Then, the Ach-stimulus was added and the fluorescence intensity was monitored in ECs and SMCs (Figure **4a, b & c**). A significant change in fluorescence intensity (p-value = 0.0008) can be appreciated already after 2.5 min of stimulation (the first possible imaging point) for ECs. The fluorescence in ECs continued to increase over a period of 15min after ACh stimulation (Figure **4d**). In SMCs no significant (p-value = 0.4) increase in fluorescence was found at any time point. The fluorescent signal can be abrogated with L-NAME or by denudation of endothelium (shown later).

### NO production can also be visualised *ex vivo* in non-precontracted murine carotid arteries and aortas with Cu _2_FL2E

**Figure 5:**
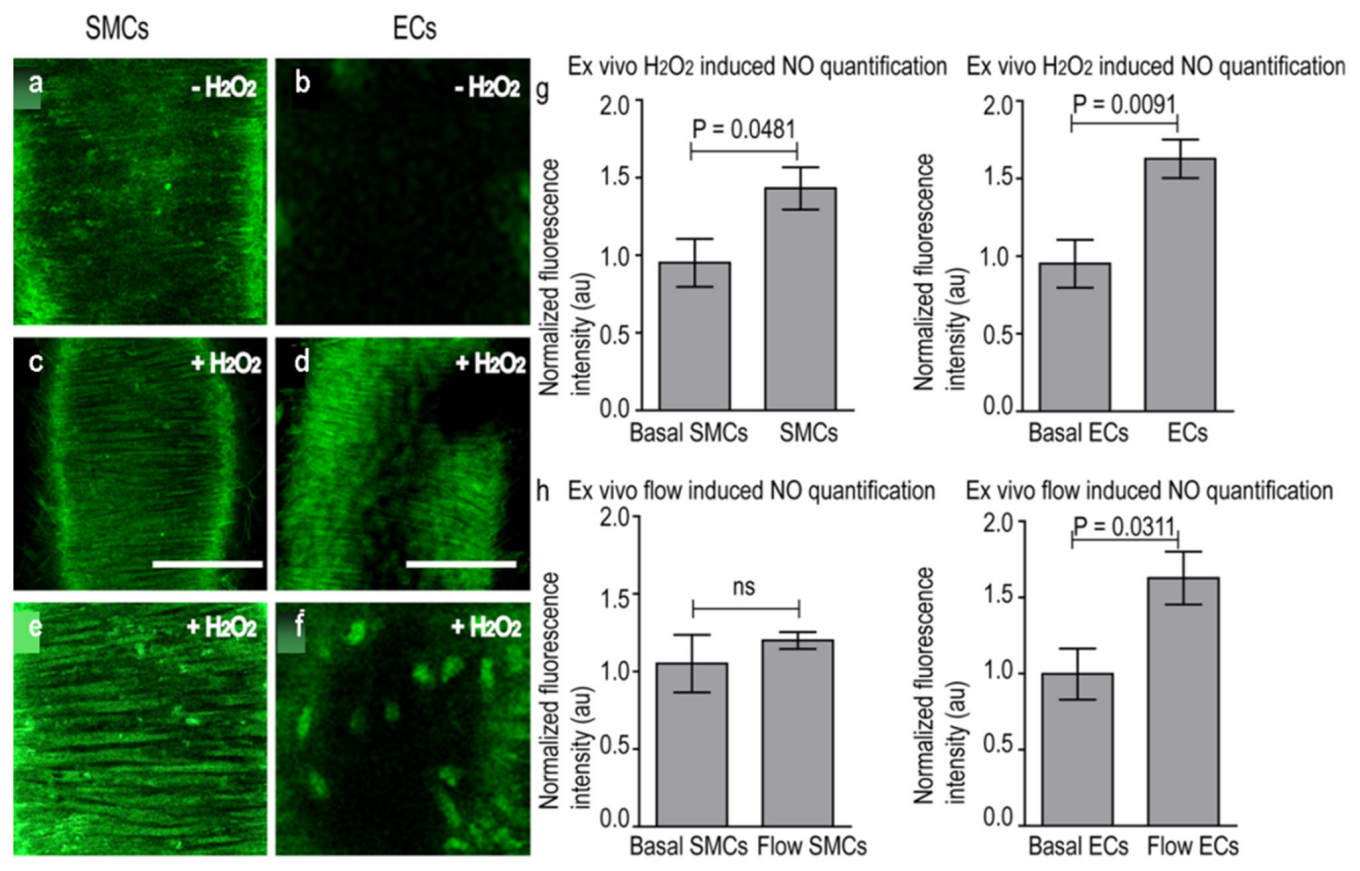
Detection of NO produced in explanted murine carotid arteries *ex* *vivo* using Cu _2_FL2E (20 µM). (**a**) & (**b**) Magnified images of vessel showing basal NO signal detected after 5 min incubation of Cu _2_FL2E without any stimulus at medial and intimal focal planes, respectively. (**c**) NO signal detected in smooth muscle cells (SMCs) and (**d**) endothelial cells (ECs) of the tissue with 5 min incubation of Cu _2_FL2Eand, subsequently 45min incubation of H_2_O_2_ (150 µM). Scale bar is 50 µm, (**e**) & (**f**) Magnified images of vessel showing NO signal detected after 5 min incubation of Cu _2_FL2E and subsequently, 45 min incubation of H_2_O_2_ (150 µM) in SMCs at medial plane and in ECs at intimal plane respectively, (**g**) Quantification of spatial distribution of fluorescence intensity as measure of NO in cells of vessel wall stimulated with H_2_O_2_ (n = 5). (**h**) Quantification of spatial distribution of fluorescence intensity as measure of NO in cells of vessel wall stimulated with flow (flow rate= 2.1 Pa, time=45min), (n = 5).

The time-profile for NO production in the vascular cells of the non-pre-contracted carotid was obtained (Figure **S1**) after stimulation with Ach and by monitoring at regular time points. With time, signal intensity increases in SMCs due to slow accumulation of NO passing from ECs. The decrease in NO signal in ECs at longer time point is probably the result of NO-probe complex saturation and subsequent bleaching. Note that interestingly the timescale of NO production is much slower and the intensity reached is much lower than in the case of pre-contracted carotid arteries (Figure 4). Therefore, in the following studies we only looked at incubation with either ACh or H_2_O_2_ for 45 min. As shown before, in a non-stimulated control artery elastin layers were visible, while ECs and SMCs were not visible. Also here, the autofluorescence (Figure **5a& b**) was independent of the presence of Cu _2_FL2E. Individual SMCs and ECs could not be identified at the starting point of analysis. After at incubation with either ACh or H_2_O_2_ for 45 min and subsequent washing and imaging, the fluorescence signal clearly reflected the presence of NO in ECs of the intimal layer and, to a lesser extent, in SMCs of the medial layer (Figure **5c-f**) for H_2_O_2_ and (Figure **S2**) for Ach. Quantification of the NO signal in vascular cells revealed that, after stimulation (here with H_2_O_2_, similar results for Ach), the fluorescence signal increased more significantly over background levels in ECs (p-value = 0.009) than in SMCs (p-value = 0.05) (Figure **5g**).

Proper labeling and focusing on the cells in different planes was demonstrated by nuclear post-staining (DAPI) in the merged images (Figure **S3**). Using L-NAME, the fluorescent signal could be abrogated (shown later in Figure 7f). Also, denudation of endothelium from the vessel results in abrogation of fluorescence in the SMCs and ECs for ACh stimuli (Figure **S4**).

Furthermore, we evaluated the ability of Cu _2_FL2E to detect more vascular and physiological stimulus, namely flow-induced [19,20,24] endogenous NO production in flow-mediated (2.1 Pa) stimulation of NO production in vessels after 45 min of flow followed by immediate imaging was observed in ECs. In this case, the increase in fluorescence over background levels for ECs was significant (p-value = 0.031), whereas the fluorescence change in SMCs was not significant (p-value = 0.5) (Figure **5h**). Again, using L-NAME, the fluorescent signal could be abrogated in these cells too (not shown).

Finally, ECs were also observed in the aorta 5 min after probe addition and subsequent 45min ACh stimulation (p-value = 0.05). However, in contrast to our findings in the carotid artery stimulated with ACh, there was no apparent and significant NO production in SMCs of aorta, (Figure **S5**). However, 3D analysis and reconstruction of the aorta shows NO-mediated relaxation occurs with an increase in luminal diameter (data not shown).

### 3D reconstruction of vessels and determination of changes in luminal diameters

On stimulation, SMCs and/or ECs become visible due to the NO-induced rise in fluorescence intensity. Therefore, the overall vessel wall volume, changes in its cellular structure, and NO production dynamics from every layer of the vessel can be observed (Video **S1**). Series of XY-images at successive depths (Z-stack, step size 0.99µm, with total 39.5µm) were collected for reconstruction of 3D images (Figure **S6**). The movie (2D stacks) and the 3D reconstructions expose the medial and intimal layer of the vessel wall. Thus, from every obtained optical section, a detailed study of delicate structures in the vessel wall was possible. The NO signal was apparent in SMCs and ECs at the medial-intimal interface of the vessel wall with good spatial resolution. Furthermore, these 3D optical sections could be used for functional analysis, including changes in luminal diameter (increase or decrease) measured from internal elastic lamina of vessels under various conditions. Unfortunately, this way of determining luminal diameters is rather slow (in contrast to the normal wide-field microscopy based methods normally used), since 3D stacks have to be reconstructed. Furthermore, to carry out 3D stacking, the vessel diameter first needs to be motionally stabilized prior to and during stimulation, in order to make images. These two factors exclude “on the fly” diameter calculations during the intensity experiments. Since changes in vascular diameter cannot be measured during the intensity experiments, end point vascular diameters of explanted carotid arteries were assessed before and after the dynamic intensity experiments. To that end, luminal diameters were obtained from XZ images after 3D reconstruction.

### NO-mediated vasomotor response of the pre-contracted and non-precontracted carotid arteries

**Figure 6:**
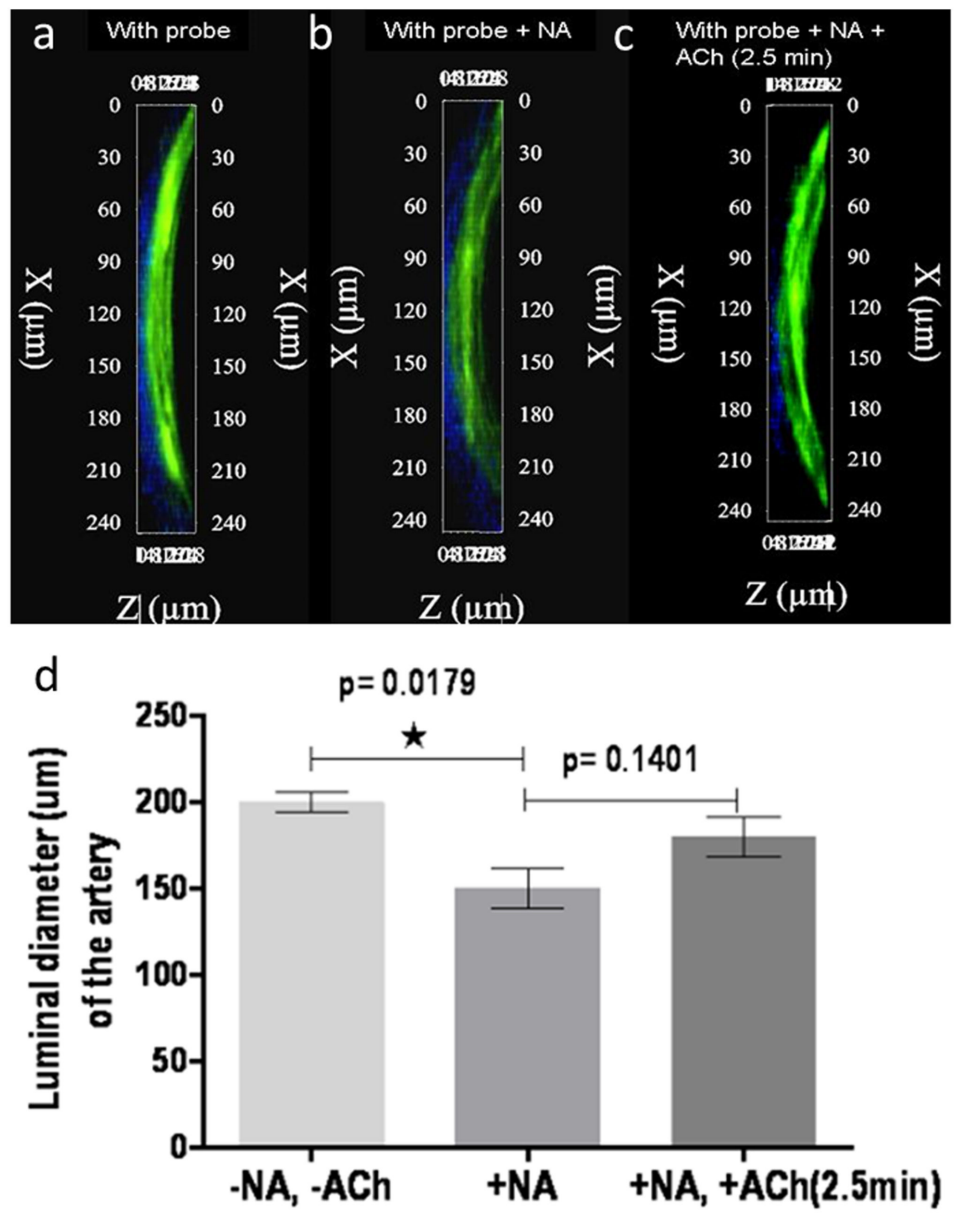
Functional imaging of NO in pre-contracted arteries. 3D reconstruction and luminal diameter measured from explanted murine carotid arteries *ex* *vivo* using Cu _2_FL2E (20 µM) (**a**) before precontraction (**b**) after precontraction with NA, (**c**) in post NA and ACh stimulation (2.5min), error bars indicate s.d. (n=3), (**d**) luminal diameter measured from arteries with conditions mentioned in a, b and c, error bars indicate s.d. (n=3).

Vasomotor function of the mounted carotid arteries was evaluated by determining change in luminal diameter from 3D constructions by adding Ach (10 µM) for the (10 µM) NA-precontracted condition. As expected, the average luminal diameter of the arteries decreased upon administration of NA with a reduced lumen diameter (p-value = 0.02) compared to non-stimulated stimulation. When ACh was added the average luminal diameter of the arteries increased slightly (p-value = 0.1) (Figure **6**).

**Figure 7:**
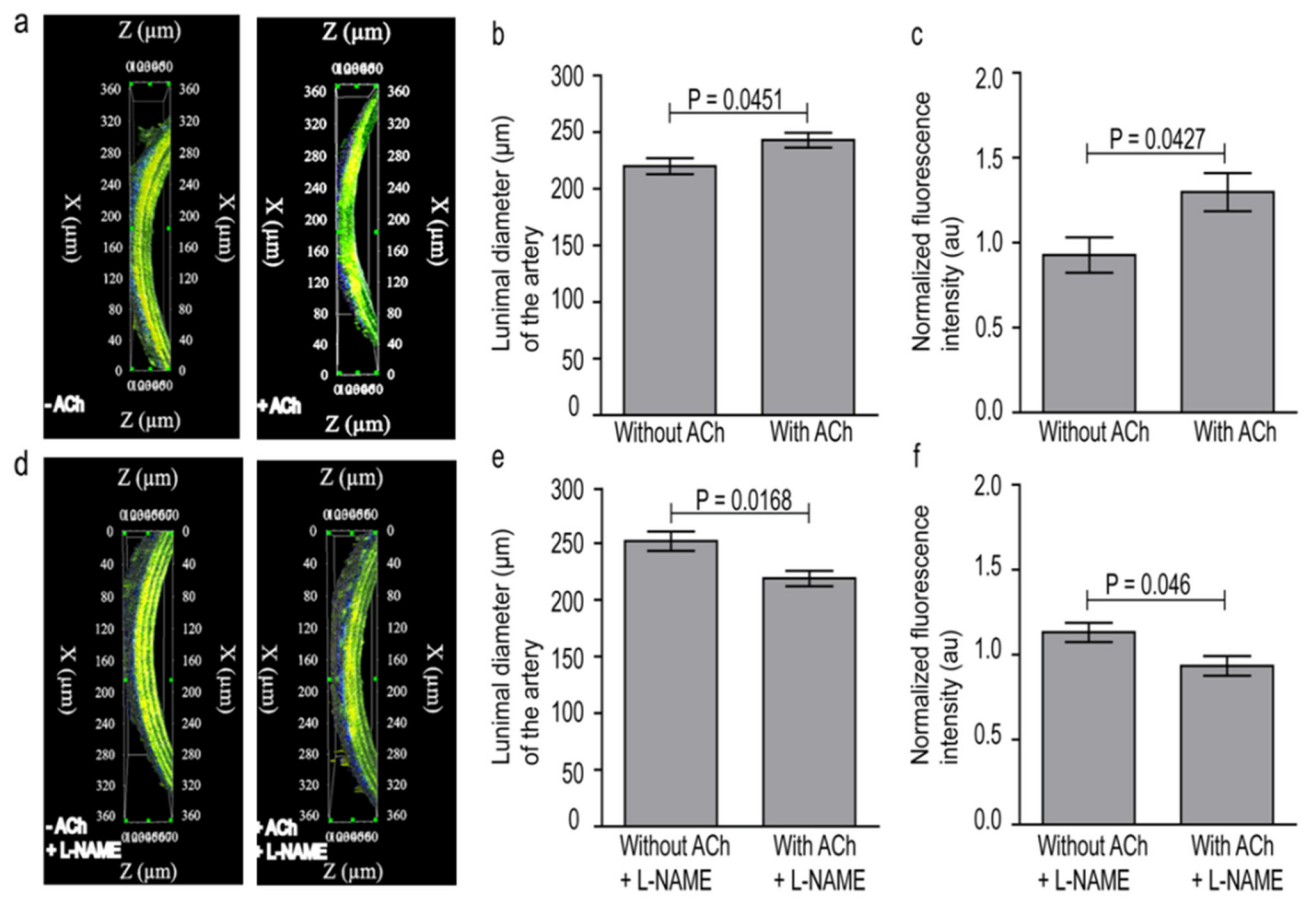
Functional imaging of NO. (**a**) 3D reconstruction of vessels with Cu _2_FL2E (20 µM) without/ with stimulus (here ACh), (**b**) luminal diameter measured from arteries with conditions mentioned in (a), (**c**) normalized fluorescence intensities of the arteries with conditions mentioned in (a), (**d**) 3D reconstruction of vessels with Cu _2_FL2E without/ with stimulus (here ACh) and also in combination with L-NAME, (**e**) luminal diameter measured from arteries with conditions mentioned in (d), (**f**) normalized fluorescence intensities of the arteries with conditions mentioned in (c), error bars indicate s.d. (n=5).

On stimulation of non-pre-contracted carotids by ACh for 45 min, the luminal diameter of the ACh-stimulated arteries increased significantly when compared with non-stimulated arteries (p-value = 0.05) (Figure **7a& b**). Also the fluorescence intensity of the ACh-stimulated arteries increased significantly when compared with non-stimulated arteries (p-value = 0.04) (Figure **7c**). In contrast, when NO synthesis in the vessel was blocked by L-NAME, luminal diameter of arteries decreased significantly (p-value = 0.02), (Figure **7d& e**) and remained almost unchanged after administration of ACh. Also the fluorescence intensity was significantly lower as compared with controls (i.e. without L-NAME) (p-value = 0.05) (Figure **7f**).

## Discussion

The present study demonstrates the feasibility of using Cu _2_FL2E as a direct, sensitive, specific, non-toxic, and rapid NO probe in vascular biology. In combination with TPLSM the probe provides a new imaging method for investigating NO biology in the vascular system. Although a variety of NO donors are available, we chose to use SNAP and maintain the experimental conditions as reported [17] in order to roughly calculate the amount of NO released accordingly. SNAP is one of the most widely used, authentic, and stable chemical sources of NO [13,17,18,25]. SNAP releases NO and other thiyl molecules almost instantaneously at a controlled rate [26]. Therefore, for short experiments, SNAP is a better choice than NONOates, since the latter have slow dissociation rate, whereas solutions of NO can dissociate to other metabolites before reaching the probe. By back calculating the NO concentration, the lower end detection limit of Cu _2_FL2E is approximately 35 nM, which is in the normal physiological range of stimulated NO levels in healthy tissue [6,17]. Thus, Cu _2_FL2E can be expected to be able to detect nano-molar concentrations of NO produced in vascular endothelial cells. We stress that further concentration calibration of the probe in solution is not useful and thus is not undertaken, because quantification in solution does not correspond with that in cell, let alone in blood vessels. Furthermore, since fluorescence intensities depend on many factors not related to concentration, microscopic intensity imaging is not an absolute but relative way of determining cellular concentration. However, penetration of the probe in the entire vessel is uniform, seen to reach from ECs to fibroblasts when artificially NO is provided in the vessel by SNAP (not shown). Also, Cu _2_FL2E demonstrated improved sensitivity and specificity for NO synthesis, combined with low cytotoxicity compared with DAF-2-DA, the most commonly used NO probe [13]. Thus, though Cu _2_FL2E could be slightly activated by other reactive oxygen species (such as H_2_O_2_), the superior specificity for NO is evident.

Because Cu(II)/Cu(I) ions can influence the rate of NO release from *S*-nitrosothiols (SNAP) or by cells associated with activated eNOS [18,25], we evaluated the influence of extra copper ions on the release of NO from SNAP. We detected no change in the response of Cu _2_FL2E and concluded that such influence is absent (not shown). Use of copper chelators, NO scavengers, and EPR measurement have additionally been used by Lim et al. [13], confirming the absence of such an influence. Also, control experiments, such as cells incubated with Cu _2_FL2E without stimulus did not exhibit significant fluorescence, indicating that Cu(I)/Cu(II) ions by themselves do not significantly increase cellular NO production. Indeed, in cells, copper released from Cu _2_FL2E is most likely scavenged rapidly by cellular components such as metallothionein or copper chaperones [13], minimizing the chance of free copper ions to influence the cellular response.

With specific chemical agents that abrogate NO synthesis, such as L-NAME, we demonstrate the specificity of the probe. Although often referred to as an inhibitor, in fact L-NAME diminishes NO production as a non-active substrate analog. Conclusively, we consider Cu _2_FL2E sensitive and specific to detect differences in physiologically relevant nanomolar concentrations of NO produced in vascular endothelial cells and arteries, which can be used to assess the contribution of NO to normal and diseased conditions.

We further show that *ex vivo* NO imaging in murine carotid arteries and murine aorta allows distinction of intimal (ECs) from medial (SMCs) NO signal. Although it is difficult to be sure about all sources of NO generation and contribution, we speculate that ECs are the prime source of NO generation in the vessel wall, as results show significant changes in fluorescence in ECs when triggered with flow or ACh. This concept is strengthened by the observation that denudation of the carotid further abrogates the signal in SMCs, as does pre-incubation with L-Name. The capacity of SMCs to generate NO (i.e. by activation of iNOS) [26,27] must also be considered (especially, by chemical triggers like H_2_O_2_). The differences in NO profiles in aorta or carotids or when induced by flow in carotid artery is subject of future study, but already indicated that the temporal occupancy profile of NO in the cells provides a general diffusion map of NO within vascular cells. The temporal NO storage in different cells plays a major role in vascular biology and pathology, because that determines NO participation in protective or pathologic role.

Along with NO-mediated vasodilation, endothelium-derived hyperpolarizing factor (EDHF) participation in relaxation of various arteries is another interesting aspect to study in this context. EDHF’s vasodilator action is of prime importance particularly when NO production is compromised. However, EDHF contributes mainly in small vessel dilation. Under our conditions, the carotids and aorta (with suitable controls) show vaso-relaxation to be primarily NO-dependent.

Quantitative differences in NO-signal *ex vivo*, as determined using Cu _2_FL2E, correlate with measurements of vasodilation or vasoconstriction, in the absence or presence of L-NAME, respectively. The results establish that Cu _2_FL2E does not crucially affect the enzymatic activity of NO synthesis, NO bioavailability, or of downstream pathways involved in SMC-relaxation. Also the correlation of NO availability and vasomotor function rules out the possibility of NO derivation from intracellular NO storage pools, as then the abrogation of NO signal with inhibitor would not necessarily abrogate vasodilation. An intriguing question yet to be answered concerns the contribution of subclasses of NO synthases (NOS) to total NO production in healthy and diseased vessels. The presented experimental methodology opens new avenues for further research on NO metabolism and its effect on vessel wall morphology and function. Direct visualization and measurement of NO would help to elucidate its role in endothelium dysfunction for diseases like atherosclerosis and hypertension. Also in complex vessel morphology such as that in atherosclerotic lesions visualization of NO will be more important.

In conclusion, this analytical method for temporal-spatial kinetics of NO synthesis allows specific detection and semi-quantification of endogenous NO production. This study provides a method to unravel the structural-functional relationship of NO in the vessel wall and the role of NO in vascular biology at an unprecedented level.

## Supporting Information

Figure S1 **The time profile for NO production in the vascular cells; NO occupancy in SMCs and ECs, of the non-pre-contracted carotid artery after stimulation with Ach (n = 5).**
(TIF)

Figure S2 **Detection of NO produced in explanted murine carotid arteries *ex vivo* using Cu _2_FL2E and Ach.** a) & b) Autofluorescence in SMCs & ECs of the tissue respectively, without Cu _2_FL2E & ACh, c) & d) Basal NO signal detected after 5 min incubation of Cu _2_FL2E (20 µM) without any stimulus in SMCs & ECs, respectively. e) & f) NO signal detected in SMCs & ECs of the tissue respectively, with 5 min incubation of Cu _2_FL2E (20 µM) and 45min incubation of ACh (10 µM). Scale bars, 50 µm.
(TIF)

Figure S3 **Labelling on the cells in different planes demonstrated by nuclear post-staining with DAPI; Magnified images of vessel showing NO signal detected in smooth muscle cells (SMCs) and endothelial cells (ECs) of the tissue with 5 min incubation of Cu _2_FL2E (20 µM) and, subsequently 45min incubation of H_2_O_2_ (150 µM), in medial and intimal focal planes respectively.** Also nuclear post-staining with DAPI shown.
(TIF)

Figure S4 **Detection of NO produced in the SMCs and ECs for ACh stimuli, in denuded endothelium; quantification of spatial distribution of fluorescence intensity as measure of NO in cells of vessel wall (n = 5).**
(TIF)

Figure S5 **Detection of NO produced in explanted murine aorta *ex vivo* using Cu _2_FL2E and Ach; quantification of spatial distribution of fluorescence intensity as measure of NO in cells of vessel wall (n = 5).**
(TIF)

Figure S6 **3D reconstruction of images from series of XY-images at successive depths (Z-stack, step size 0.99µm, with total 39.5µm).** a) 3D reconstruction of a section of the vessel showing “spindle shaped” smooth muscle cell and endothelial cell (*several indicated by red circles*) alignment with respect to the direction of flow, b) 3D reconstruction of the intimal side of the vessel exposing smooth muscle cells and endothelial cells at the media-intima interface to assess the structure of the cells in relation to variable NO release, c) 3D reconstruction of the adventitial side of the vessel showing thin elastin fibres and fibroblasts at the adventitia-media interface.
(TIF)

Video S1 **NO production dynamics from every layer of the vessel (the overall vessel wall volume, changes in its cellular structure); direct visualization of NO signal in SMCs at the medial layer and in ECs at the intimal layer of the vessel wall.** In the movie, part of the murine carotid artery can be observed aligned horizontally that has been incubated with Cu _2_FL2E stimulated with ACh, then scanning using TPLSM takes place from the adventitial side to luminal side. Fibroblasts along with collagen (in blue) can be seen at the adventitia, with progression to medial layer, “spindle shaped” SMCs (green) aligned vertically becomes apparent due to NO signal and following to intimal side, ECs aligned horizontally can be seen to produce NO signal (in green). In this movie, z-stack of individual 42 sections has been taken at a depth of 39.5 µm, with each step size of 0.99 µm. The scanning speed was 100Hz with optical zoom of 3.3.
(AVI)
