# Supplementary figures and images for: Specific Visualization of Nitric Oxide in the Vasculature with Two-Photon Microscopy Using a Copper Based Fluorescent Probe
Source: PLoS One. 2013 Sep 23;8(9):e75331. doi: 10.1371/journal.pone.0075331 (PMC3781046; doi:10.1371/journal.pone.0075331)

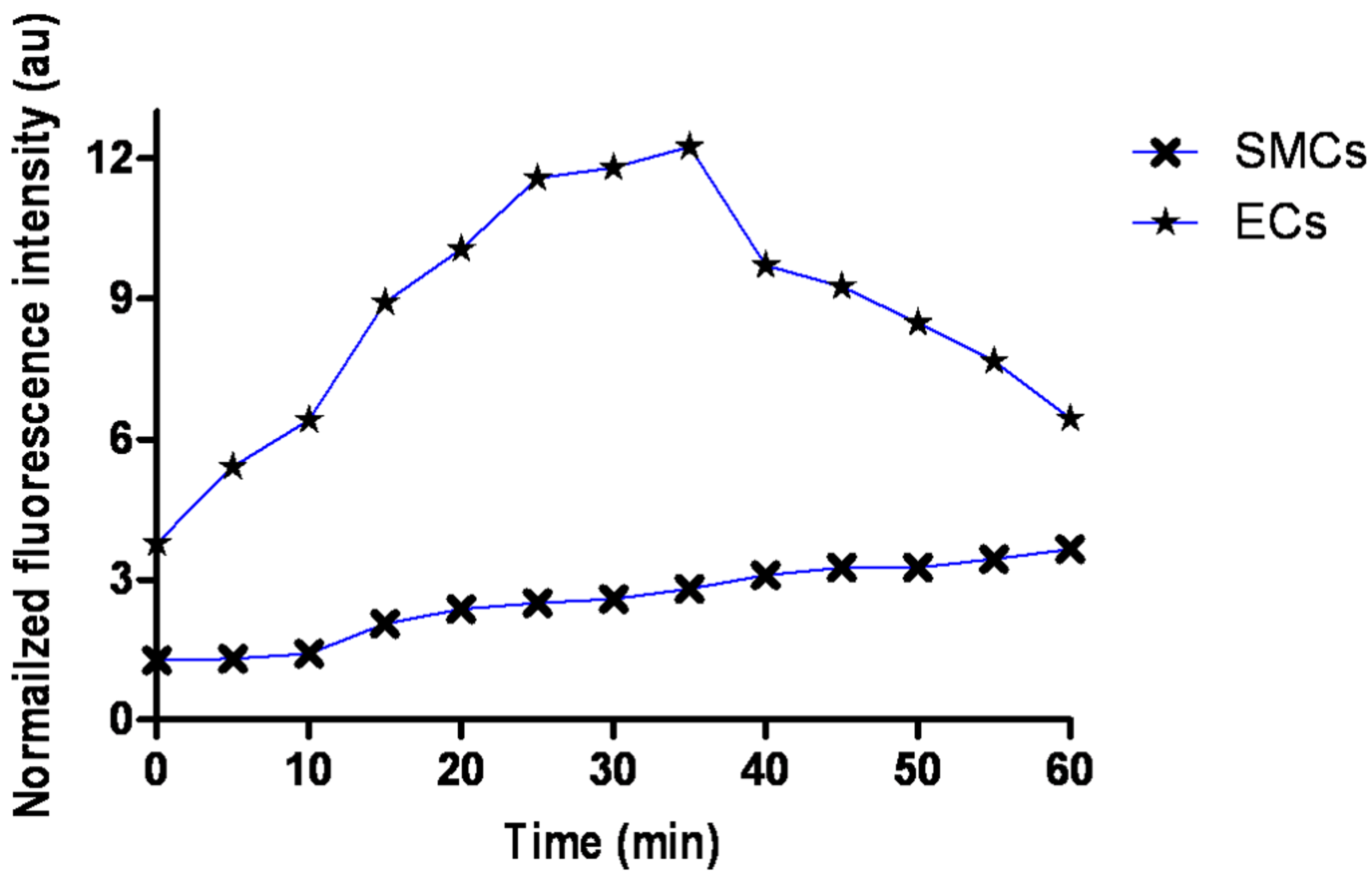

Supplement: Figure S1 — The time profile for NO production in the vascular cells; NO occupancy in SMCs and ECs, of the non-pre-contracted carotid artery after stimulation with Ach (n = 5). (TIF) [file pone.0075331.s001.tif]

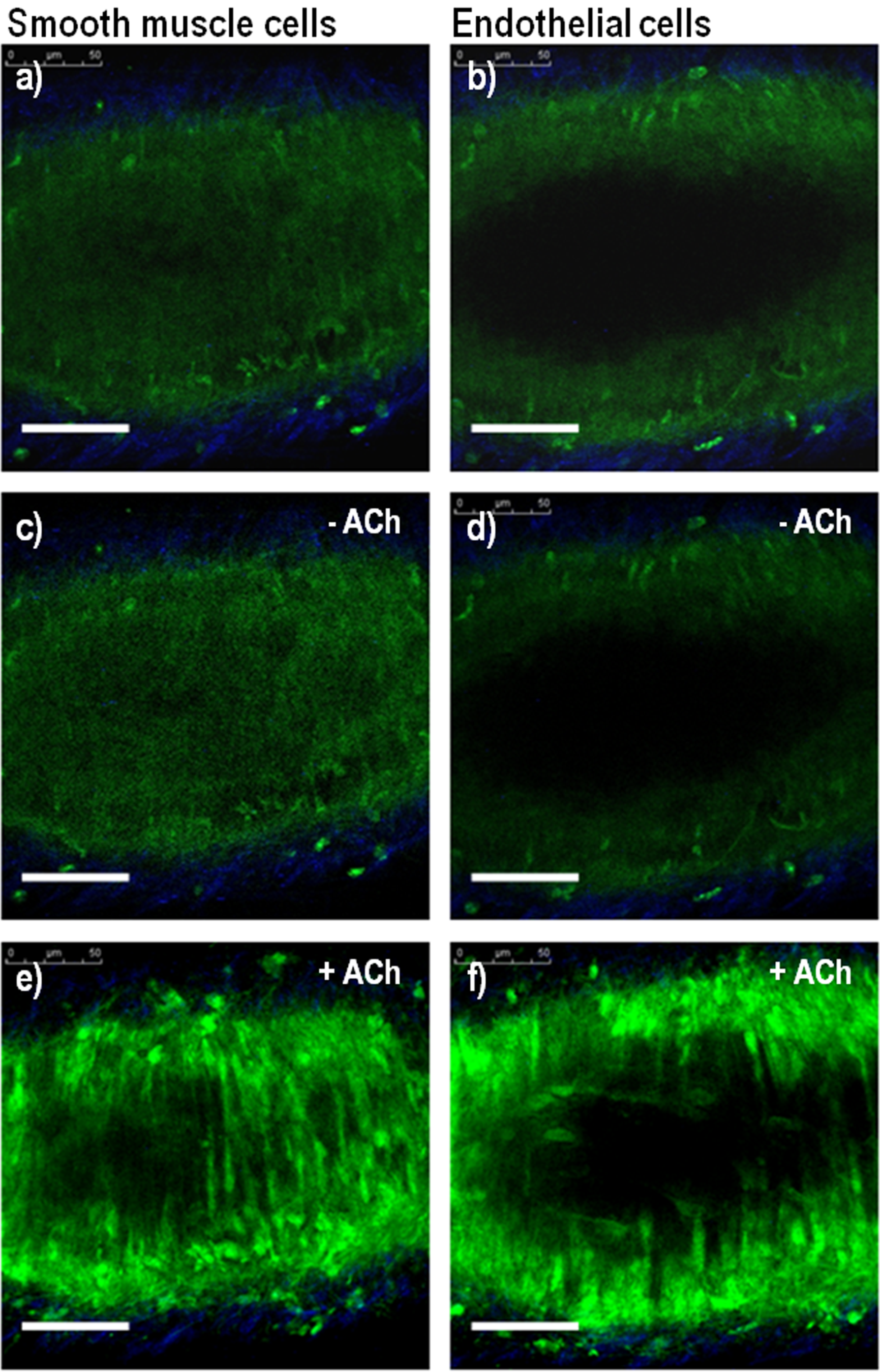

Supplement: Figure S2 — Detection of NO produced in explanted murine carotid arteries ex vivo using Cu 2FL2E and Ach. a) & b) Autofluorescence in SMCs & ECs of the tissue respectively, without Cu 2FL2E & ACh, c) & d) Basal NO signal detected after 5 min incubation of Cu 2FL2E (20 µM) without any stimulus in SMCs & ECs, respectively. e) & f) NO signal detected in SMCs & ECs of the tissue respectively, with 5 min incubation of Cu 2FL2E (20 µM) and 45min incubation of ACh (10 µM). Scale bars, 50 µm. (TIF) [file pone.0075331.s002.tif]

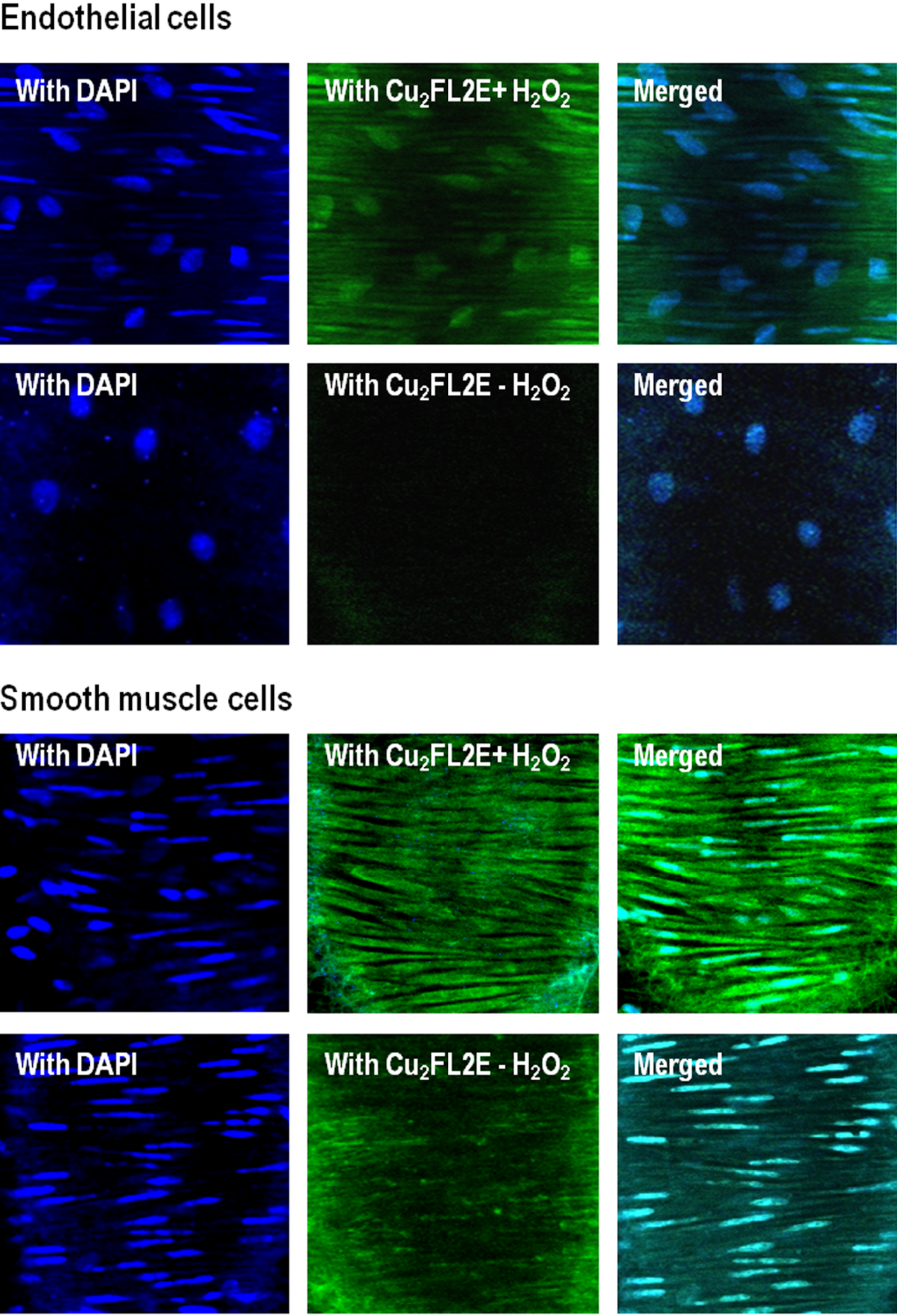

Supplement: Figure S3 — Labelling on the cells in different planes demonstrated by nuclear post-staining with DAPI; Magnified images of vessel showing NO signal detected in smooth muscle cells (SMCs) and endothelial cells (ECs) of the tissue with 5 min incubation of Cu 2FL2E (20 µM) and, subsequently 45min incubation of H2O2 (150 µM), in medial and intimal focal planes respectively. Also nuclear post-staining with DAPI shown. (TIF) [file pone.0075331.s003.tif]

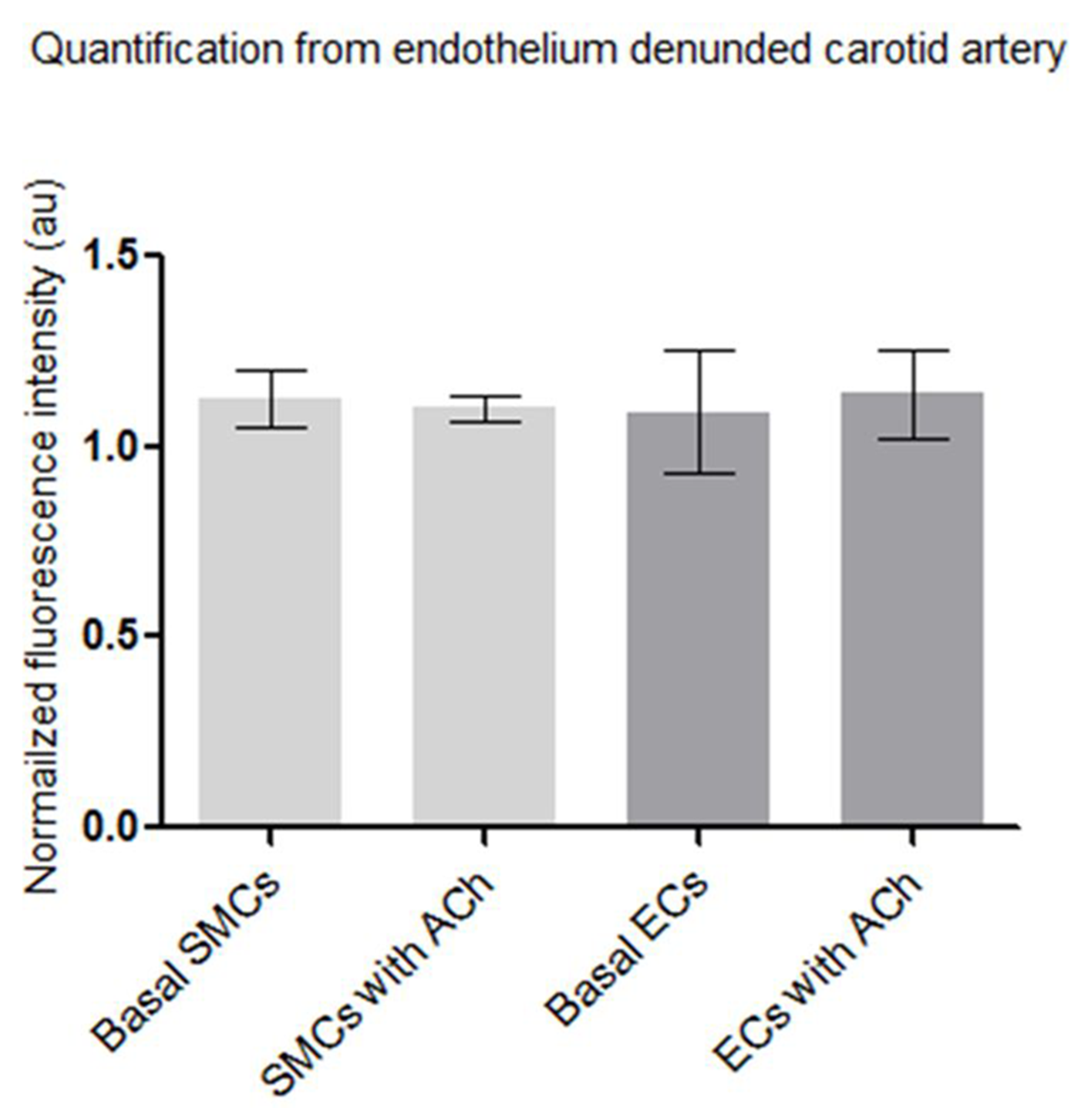

Supplement: Figure S4 — Detection of NO produced in the SMCs and ECs for ACh stimuli, in denuded endothelium; quantification of spatial distribution of fluorescence intensity as measure of NO in cells of vessel wall (n = 5). (TIF) [file pone.0075331.s004.tif]

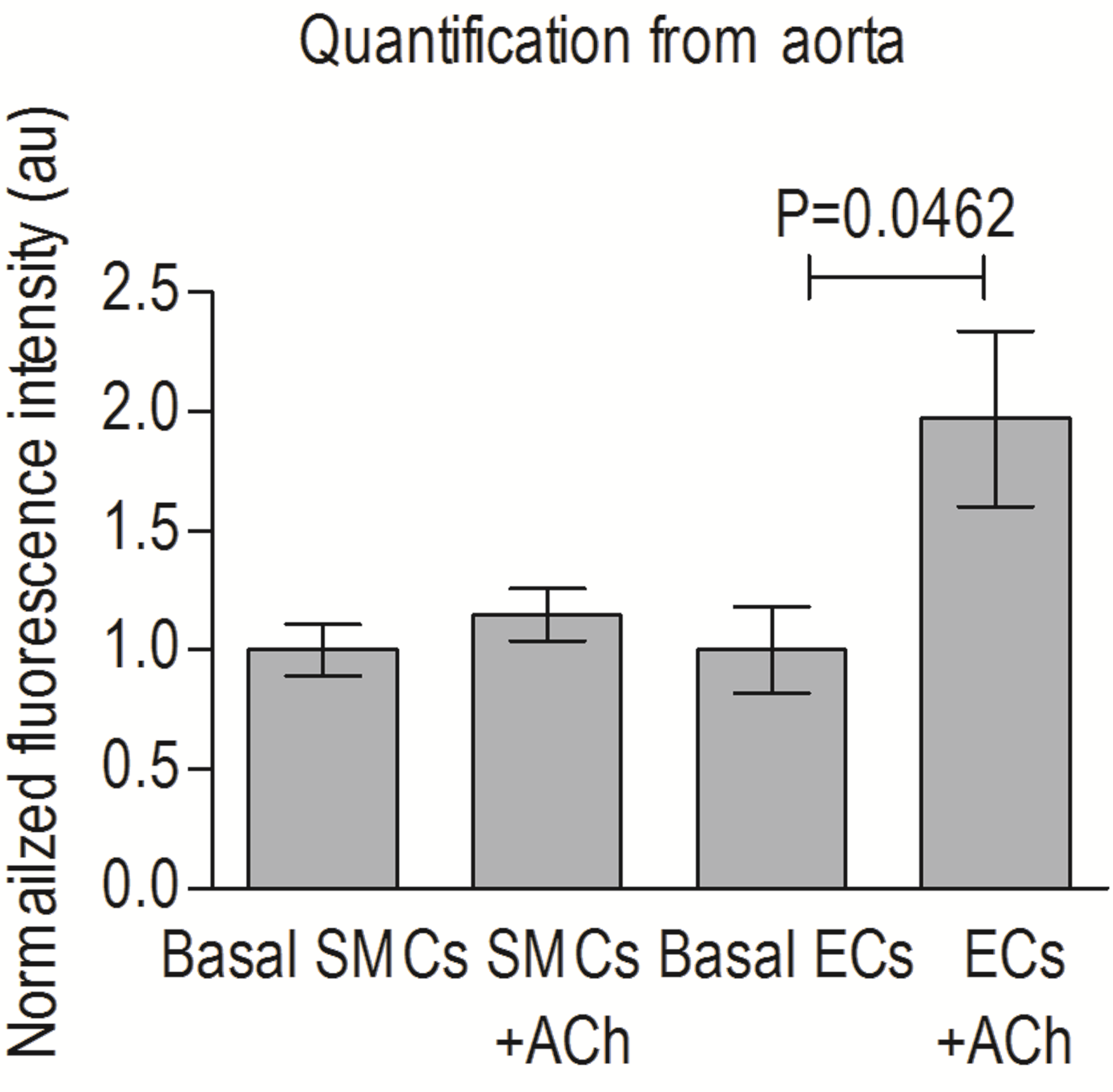

Supplement: Figure S5 — Detection of NO produced in explanted murine aorta ex vivo using Cu 2FL2E and Ach; quantification of spatial distribution of fluorescence intensity as measure of NO in cells of vessel wall (n = 5). (TIF) [file pone.0075331.s005.tif]

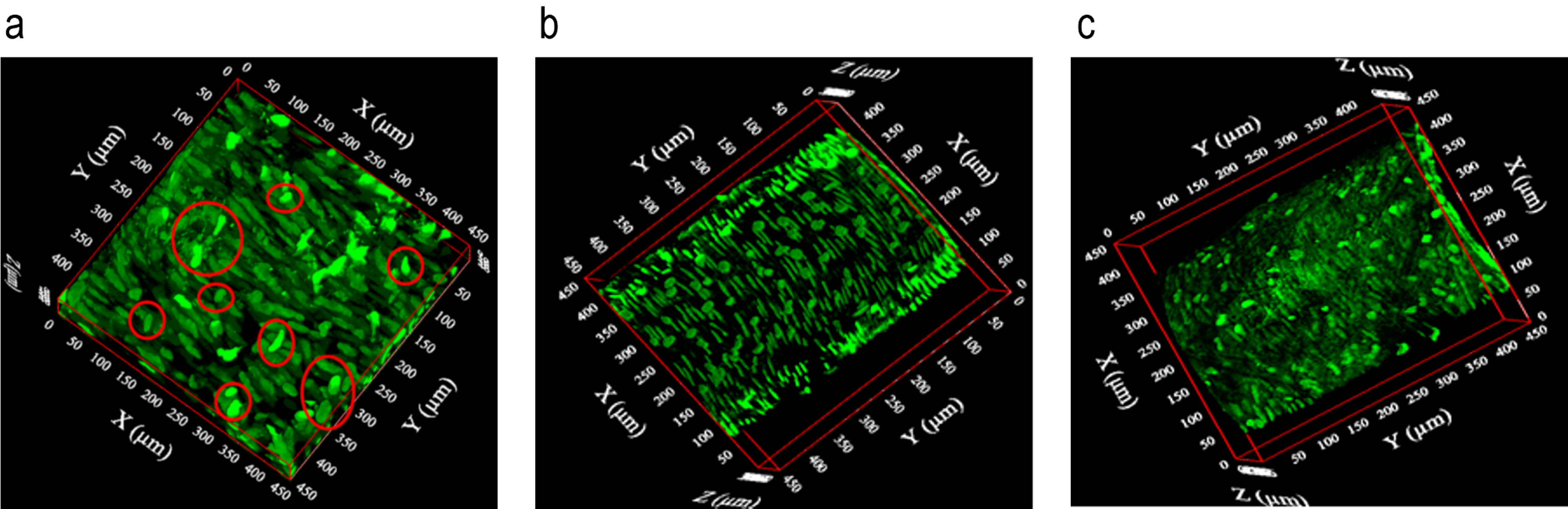

Supplement: Figure S6 — 3D reconstruction of images from series of XY-images at successive depths (Z-stack, step size 0.99µm, with total 39.5µm). a) 3D reconstruction of a section of the vessel showing “spindle shaped” smooth muscle cell and endothelial cell (several indicated by red circles) alignment with respect to the direction of flow, b) 3D reconstruction of the intimal side of the vessel exposing smooth muscle cells and endothelial cells at the media-intima interface to assess the structure of the cells in relation to variable NO release, c) 3D reconstruction of the adventitial side of the vessel showing thin elastin fibres and fibroblasts at the adventitia-media interface. (TIF) [file pone.0075331.s006.tif]
